# SARS-CoV-2-induced venous thromboembolism in a patient with VEXAS syndrome

**DOI:** 10.1093/rap/rkad012

**Published:** 2023-01-20

**Authors:** Bruno Lucchino, Lorenzo Delfino, Giuseppe Patuzzo, Annacarla Finucci, Francesco Ghellere, Maria Elena Bortolotti, Sara Lombardi

**Affiliations:** General Medicine Unit, Girolamo Fracastoro Hospital, AULSS9 Scaligera, San Bonifacio, Verona, Italy; General Medicine Unit, Girolamo Fracastoro Hospital, AULSS9 Scaligera, San Bonifacio, Verona, Italy; Emergency Unit, Girolamo Fracastoro Hospital, AULSS9 Scaligera, San Bonifacio, Verona, Italy; General Medicine Unit, Girolamo Fracastoro Hospital, AULSS9 Scaligera, San Bonifacio, Verona, Italy; General Medicine Unit, Girolamo Fracastoro Hospital, AULSS9 Scaligera, San Bonifacio, Verona, Italy; General Medicine Unit, Girolamo Fracastoro Hospital, AULSS9 Scaligera, San Bonifacio, Verona, Italy; General Medicine Unit, Girolamo Fracastoro Hospital, AULSS9 Scaligera, San Bonifacio, Verona, Italy

Key message

- SARS-CoV-2 infection in VEXAS syndrome can cause thromboembolism, which responds to combined immunosuppressive and anticoagulant treatment.

Dear Editor, We report the case of a 75 year-old man, diagnosed with VEXAS (vacuoles, E1 enzyme, X-linked, autoinflammatory, somatic) syndrome, who developed a thromboembolic complication following SARS-CoV-2 infection. The disease manifested with fever, subcutaneous erythema nodosum-like lesions, ear chondritis, arthritis, episcleritis and oral aphthosis, as previously described [1]. The patient presented macrocytic anaemia, secondary to bone marrow myeloerythroid dysplasia, and elevated inflammatory markers. The diagnosis was confirmed by the demonstration of *UBA1* gene somatic mutation (A>G, Met41Val) on peripheral blood cells.

The condition was refractory to multiple immunosuppressive drugs, requiring high-dose prednisone for symptom management. Accordingly, tocilizumab was prescribed, but at the end of April 2022, before treatment beginning, the patient presented to the emergency department for cough and dyspnoea. An antigenic test for SARS-CoV-2 was positive. A thoracic CT scan demonstrated ground-glass opacification in both superior lung lobes, without evidence of pulmonary embolism. The patient was admitted to our department, and an antiviral treatment with remdesivir was started, together with low molecular weight heparin thromboprophylaxis. After a rapid clinical improvement, the patient was discharged with a further 14-day course of thromboprophylaxis. Two weeks later, the fever and subcutaneous lesions relapsed, requiring an increasing dose of prednisone for disease control, up to 30 mg/day.

In June 2022, the patient was re-admitted to our department for the development of left lower limb oedema. A CT scan showed a left femoral vein thrombosis and a pulmonary embolus in the segmental posterior basal branch of the right pulmonary artery (Fig. 1). The D-Dimer level was 3.34 mg/l, and CRP was slightly elevated (15.9 mg/l) (Fig. 1). C-protein, S-protein, anti-thrombin III and homocysteine levels were normal. Factor II and V mutations, anti-cardiolipin, anti-β2-glicoprotein antibodies and LA were negative. Fondaparinux treatment was initiated, simultaneously with s.c. tocilizumab treatment. A dramatic improvement was observed, with resolution of the limb oedema. The s.c. anticoagulant was promptly switched to oral apixaban 5 mg twice daily, and the patient was discharged. During the follow-up, the patient was in complete remission on tocilizumab treatment, with normalization of inflammatory markers, allowing prednisone taper. Furthermore, no thromboembolic recurrences were observed, and the patient was maintained on apixaban treatment indefinitely.

**Figure 1.**
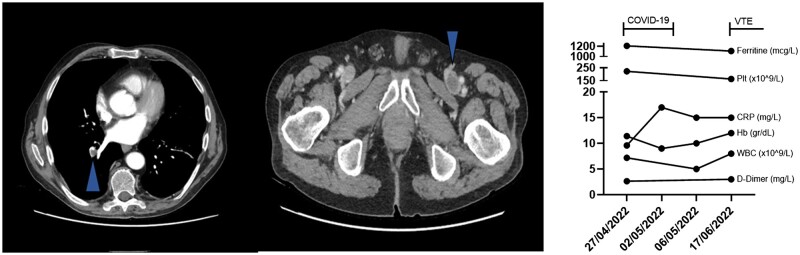
CT scan of the patient and time course of laboratory parameters. Arrowheads indicate thrombotic material in the left femoral vein and in the segmental posterior basal branch of the right pulmonary artery. Hb: Haemoglobin; Plt: Platelets; VTE: venous thromboembolism; WBC: white blood cells

Thromboembolism is a major complication of VEXAS syndrome, occurring in ∼35–56% of patients and manifesting mostly as venous thromboembolism (VTE) rather than arterial thrombosis [2, 3]. UBA1 loss of function, resulting in an impaired ubiquitination, triggers an unfolded protein response in myeloid cells, resulting in hyperproduction of cytokines and systemic inflammation. Inflammatory cytokines, including TNF-α and IL-6, are involved in induction of a pro-thrombotic state, through their actions on leucocytes, platelets and endothelial cells [2]. Increased levels of coagulation factors VIII and IX have been reported in VEXAS patients experiencing VTE. Moreover, ∼50% of VEXAS patients manifesting with VTE feature a positive LA [3]. Lastly, it is well known that systemic glucocorticoids can increase VTE risk [4], suggesting multiple mechanisms contributing to VTE in this condition. Coronavirus disease 2019 (COVID-19) is frequently complicated by VTE, with an overall prevalence of 14.1% [5]. The inflammatory response to SARS-CoV-2 infection, termed a cytokine storm, is central in the pathogenesis of COVID-19 manifestations, including VTE, justifying the use of several anti-rheumatic agents since the beginning of the pandemic [5, 6]. The IL-6 inhibitor tocilizumab, which has been widely used during COVID-19 pandemic, represented the agent of choice in our patient for the potential benefits on both VEXAS manifestations and the COVID-19 cytokine storm.

The case of our patient underlines the potential consequences of SARS-CoV-2 infection in VEXAS patients. Onset of VEXAS syndrome has been described after SARS-CoV-2 infection. Both virally induced hyperproduction of cytokines and an inhibitory effect of SARS-CoV-2 on the function of UBA1 might contribute to the onset or exacerbation of the disease, as occurred in our patient [7]. In contrast, there are no reports of SARS-CoV-2 infection precipitating a VTE in VEXAS syndrome, as strongly suggested by the time line of our patient. National Institute for Health and Care Excellence (NICE) guidelines for COVID-19 management recommend a thromboprophylaxis with low molecular weight heparin for adults with COVID-19, which should be continued for ≥7 days, including after discharge [8]. However, such a short course thromboprophylaxis might be insufficient to prevent VTE in VEXAS syndrome, especially in the presence of active inflammatory manifestations and high-dose CSs use, as observed in our patient. This would suggest a precautionary extension of prophylaxis, at least until inflammation is under control, if allowed by the patient’s condition.

To date, there are no specific indications for VTE treatment in VEXAS syndrome. Combining immunosuppressive treatment with tocilizumab and the standard anticoagulation was successful, indicating the inflammatory process as a driver of thrombotic manifestations. Indeed, an analogous strategy (using, however, HCQ rather than tocilizumab) has been reported by Oo *et al.* [2], with a similar positive outcome. Both patients have been switched promptly to oral apixaban without VTE recurrence, suggesting that direct oral anticoagulants might be effective in VEXAS thrombosis in the absence of aPL. Although the appropriate duration of anticoagulation in VEXAS patients who develop a VTE event is unknown, it is our opinion that long-term anticoagulation should be maintained, in order to prevent the potentially devastating consequences of a VTE relapse.

In conclusion, SARS-CoV-2 infection might expose VEXAS patients to disease exacerbations and contribute to VTE. Accordingly, SARS-CoV-2 infection prevention strategies and information for patients should be provided. The anecdotal efficacy of immunosuppression in combination with anticoagulation might be promising and deserves to be assessed in large cohorts.

## Data Availability

Data are available upon reasonable request by any qualified researchers who engage in rigorous, independent scientific research, and will be provided following review and approval of a research proposal and Statistical Analysis Plan (SAP) and execution of a Data Sharing Agreement (DSA). All data relevant to the study are included in the article.
